# CXCL7-Mediated Stimulation of Lymphangiogenic Factors VEGF-C, VEGF-D in Human Breast Cancer Cells

**DOI:** 10.1155/2010/939407

**Published:** 2010-06-22

**Authors:** Minghuan Yu, Richard Berk, Mary Ann Kosir

**Affiliations:** ^1^Department of Surgery, Wayne State University, Detroit, MI 48201, USA; ^2^Department of Immunology and Microbiology, Wayne State University, Detroit, MI 48201, USA; ^3^Surgical Service, John D. Dingell VA Medical Center, Detroit, MI 48201, USA; ^4^Breast Biology Program, Karmanos Cancer Institute, Detroit, MI 48201, USA

## Abstract

Increased expression of lymphangiogenesis factors VEGF-C/D and heparanase has been correlated with the invasion of cancer. Furthermore, chemokines may modify matrix to facilitate metastasis, and they are associated with VEGF-C and heparanase. The chemokine CXCL7 binds heparin and the G-protein-linked receptor CXCR2. We investigated the effect of CXCR2 blockade on the expression of VEGF-C/D, heparanase, and on invasion. CXCL7 siRNA and a specific antagonist of CXCR2 (SB225002) were used to treat CXCL7 stably transfected MCF10AT cells. Matrigel invasion assays were performed. VEGF-C/D expression and secretion were determined by real-time PCR and ELISA assay, and heparanase activity was quantified by ELISA. SB225002 blocked VEGF-C/D expression and secretion (*P* < .01). CXCL7 siRNA knockdown decreased heparanase (*P* < .01). Both SB225002 and CXCL7 siRNA reduced the Matrigel invasion (*P* < .01). The MAP kinase signaling pathway was not involved. The CXCL7/CXCR2 axis is important for cell invasion and the expression of VEGF-C/D and heparanase, all linked to invasion.

## 1. Introduction

Breast cancer is the most common malignancy in women, and metastasis is the number one cause of mortality in breast cancer [1]. Breast cancer treatments that target the steps of metastasis are needed. An important part of metastasis is invasion [2, 3]. Invasion depends in part on degradation of extracellular matrix (ECM) and interaction with molecules formed in the process. Recently, increasing attention has been paid to chemokines that may modify breast cancer cells and the surrounding matrix to facilitate metastasis. Furthermore, there is evidence that chemokines, VEGF-C, and heparanase are interrelated in the process of invasion [4–6]. Increased expression of the lymphangiogenesis factors and heparanase has been correlated with progressive disease in certain cancers [6]. Peritumoral lymphangiogenesis involves the secretion of specific glycoproteins designated vascular endothelial growth factor C (VEGF-C) and (VEGF-D) that act on lymphatic endothelium, and are components of an established signaling system for tumor lymphangiogenesis [7]. Increased lymph node metastases are correlated with increased expression of VEGF-D and VEGFR3 by immunohistochemistry in invasive breast cancer [8]. 

Heparanase is an endo-*β*-glucuronidase that cleaves heparan sulfate (HS) side chains of heparin sulfate proteoglycan (HSPG). Heparanase activity has been correlated with cell invasion associated with breast cancer metastasis, a consequence of structural modification of HS that alters the extracellular matrix [9, 10]. However, classical mammalian heparanase is an intracellular enzyme and is not specific to metastatic sites [11–13]. Therefore, another source of heparanase may come from chemokines which are secreted.

Chemokines are a family of small molecular weight proteins. CXCL7 is a member of the CXC subfamily of chemokines which can be further subdivided on the basis of the presence of the tripeptide motif glutamate-leucine-arginine (ELR). All ELR^+^-CXC chemokines act through CXC chemokine receptor type1 or type2 (CXCR1 and CXCR2) [14]. CXCL7, has dual functions of heparin binding and is a ligand to the G-protein linked receptor CXCR2 [15].

 Until recently, CXCL7 gene expression was thought to be restricted to cells within the megakaryocytic lineage [16, 17], as well as to neutrophils and lymphocytes. Recent reports have suggested that other cell types may produce this chemokine as well [18–23]. Despite studies on increased expression of CXCR2 in breast cancer, reports on CXCL7 in breast cancer are limited.

Earlier studies from our laboratory have shown that the malignant breast cancer cells express more CXCL7 than premalignant MCF10AT cells. CXCL7-transfected MCF10AT breast cells have much higher heparanase activity than premalignant MCF10AT cells. CXCL7-transfected MCF10AT cells are as invasive as malignant breast cancer cells [24]. In addition, the expression of VEGF C and D is increased in these transfectants [5].

 Heparanase and the lymphangiogenesis factors VEGF-C and VEGF-D are two important markers closely related to the metastasic capabilty of breast cancer. In this paper, we hypothesize that CXCL7 increases the expression of VEGF-C, VEGF-D, and heparanase, and increases cell invasion via CXCR2 signaling, all linked to tumor lymphangiogenesis and metastasis.

## 2. Methods

### 2.1. Cell Culture and Plasmid Stable Transfection

The MCF10AT cells [25] were cultured in Dulbecco's Modified Eagle's Medium (DMEM)/F 12 (1 : 1) containing 5% horse serum, supplemented with 10 *μ*g/ml bovine insulin, EGF (20 ng/ml), hydrocortisone (0.5 *μ*g/ml), and cholera toxin (100 ng/ml). Cells were cultured at 37°C in a 5% CO_2_ atmosphere. All cell culture reagents were obtained from Gibco (Grand Island, NY). Approximately, 1 × 10^6^ cells/well were plated in 6-well plates in medium containing 5% horse serum to grow overnight to 60%–70% confluency. Transfection of the plasmid was performed by using Lipofectamine 2000 (Invitrogen, USA). The cells were divided into blank control group, negative control group, and the test group. Only Lipofectamine 2000 was used for transfection in the blank control group. Plasmid _P_EGFP-N1 was used for transfection in the negative control group. Plasmid _P_EGFP-N1-CXCL7 was used for transfection in the test group. The cells were transfected with the mixture of plasmid and Lipofectamine 2000 (1: 3) in 2 ml serum-free medium. At 24 hours after transfection, the medium was replaced by normal medium containing 5% FBS and antibiotics up to 72 hours post-transfection. Since the MCF10AT cells were transfected cells, G418 could not be used for the selection of the stably transfected cell line. Therefore, we submitted the cells for the flow cytometry sorting by using the EGFP antibody. The transfected cells were picked out for subculture. For sh-RNA plasmid transfection we used the same method the described above. The sh-PPBP plasmids were purchased from ORIGENE (Rockville, MD).

### 2.2. In Vitro Invasion Assays

Briefly, BioCoat Matrigel invasion chambers (Becton-Dickinson, Bedford, MA) were rehydrated according to the manufacturer's instructions. Cell suspensions (2.5 × 10^5^ cells per 2 ml serum-free medium) were added to the top chamber, and complete medium in the lower chamber. For control, inserts without Matrigel were used. The cells were allowed to invade the Matrigel at 37°C in 5% CO_2_ for 48 hours. The noninvading cells on the upper surface of membrane were removed from the chamber by gentle scrubbing with a cotton swab, and the invading cells on the lower surface of the membrane were stained with the Quick-Diff stain kit (Becton-Dickinson). After two washes with water, the chambers were allowed to air dry. Membranes were mounted on glass slides and counted manually under a light microscope. Cells were counted in five high power fields (40x magnification). The number of invading cells was expressed as a percentage by the following: the mean number of the cells invading through the Matrigel insert membrane divided by the mean number of cells migrating under control insert membrane conditions multiplied by 100. All assays were performed in triplicate.

### 2.3. Quantitative Real-Time PCR

RNA was extracted using the RNeasy Mini Kit (catalog no. 74,804; Qiagen, Valencia, CA), including treatment with DNase I to prevent genomic DNA contamination using RURBO DNA-free Kit (catalog no. AM1907; Ambion, Foster City, CA) according to the manufacturer's instructions. Total RNA (two micrograms) was reverse-transcribed to cDNA by using the SuperScript III First-Strand Synthesis System (catalog no. 18080-051; Invitrogen, Carlsbad, CA), according to the manufacturer's protocol. Three replicate samples were used for the three cell lines. The primer sequences used for the reactions are in Table 1 along with expected products and GenBank accession numbers. The thermocycler parameters were as follows: an initial step at 95°C for 10 min., 40 cycles of 95°C for 20 sec., 58°C for 30 sec., and 72°C for 20 seconds. The cycle threshold values were used to calculate the normalized expression of VEGFC/D against *β*-actin. qRT-PCR was performed in ABI 7500 Sequence Detection System using a SYBR Green detection system (catalog no. 170- 8880; Bio-Rad Laboratories). By identifying the threshold cycle (C_T_) for expression of mRNA, the ΔC_T_ for VEGF-C/D was calculated and compared to vector transfected control cells; CXCL7 transfected cells and SB225002-treated CXCL7 transfected cells. The ΔC_T_ was converted into a ratio (target A/target B = 2^−ΔCT^) describing the comparison of the relative expression of VEGF-C/D (target A) to *β*-actin (target B) for each of the lines. The Delta-delta model was used for comparison of relative expression RT-PCR results for VEGF-C/D.

### 2.4. Protein Quantification

VEGF-C and VEGF-D protein concentrations in conditioned media were measured by enzyme-linked immunosorbent assay (ELISA) using human VEGF-C or VEGF-D ELISA Development System (catalog no. DVEC 00 and DVED00, R&D Systems, Minneapolis, MN). Measurements were done at least in duplicate for 2 dilutions. The optical density at 570 nm and 450 nm was determined for each well using the plate reader. Then the reading at 570 nm was subtracted from the reading at 450 nm for each well.

### 2.5. Heparanase Activity Measurement

The protein concentration of the serum-free conditioned medium was measured by the Bradford assay (BioRad, Richmond, CA, USA). The heparanase activity of the conditioned medium was assessed as heparan sulfate degrading enzyme activity using the Heparan Degrading Enzyme Assay Kit (catalog no. MK412, Takara, Ootsu, Japan). The duration of a series of assays was 100 min., including 45 min. of enzyme degradation reaction. One unit of heparanase activity defined the activity which degraded 0.063 ng of biotinylated heparin sulfate in 1 min. at 37°C and pH 5.8. The detection limit of this assay was 0.1 U/mL. Each value of heparanase activity was normalized by protein concentration (U/g protein).

### 2.6. Western Blotting

Cells were processed for protein extraction and western blotting using standard procedures. Briefly, the cells were harvested in PBS, counted, and lysed in the RIPA buffer (catalog no. R0278, SIGMA, St. Louis, MO) with protease inhibitor cocktail (catalog no. P8340, SIGMA, St. Louis, MO), and samples were kept at 4°C. Protein concentration was determined for all samples using the Bio-Rad protein assay (catalog no. 500-0006, Bio-Rad, Richmond, CA). The equal-volume samples (50 *μ*g) were separated by SDS-PAGE on a 10% polyacrylamide gel and transferred onto nitrocellulose membrane (catalog no. 162-0114, Bio-Rad, Richmond, CA) using transfer tank. Immunodetection was performed using pERK1/2 (catalog no. 9101, Cell Signaling Technology) and ERK1/2 (catalog no.9102, Cell Signaling Technology), then developed by ECL (catalog no. PRN 2106, GE Healthcare, Waukesha, WI) and was photodocumented.

### 2.7. Statistical Analysis

Results are expressed as mean ± standard error of the mean (SEM), unless indicated otherwise. For statistical analysis, one-way ANOVA was used, and significance was defined as *P* < .05. Graphs were generated using GraphPad Prism (GraphPad Software, San Diego, CA).

## 3. Results

### 3.1. CXCR2 Antagonist SB225002 Reduced Invasion of Matrigel by Stably CXCL7-Transfected MCF10AT Cells

**Figure 1:**
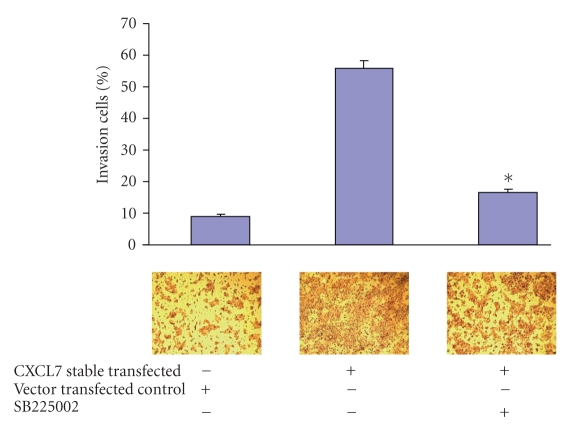
CXCR2 antagonist SB225005 reduced the invasion of MCF10AT stably transfected with CXCL7 using a BD BioCoat Matrigel invasion assay (6-well plates), 1.25 x 10^5^ cells/ml were inoculated onto the membrane in serum-free medium. For the blocking experiment, there was serum-free medium containing SB225002 (1.1 *μ*M) in the upper chamber. The lower chamber contained complete medium, and the control membrane did not have Matrigel by which to measure the migration of cells. After 48 hours, membranes were fixed, stained, and photographed, then the percent invasion was determined. Triplicate assays were done. Results are reported per cell line as percent invasion ± (mean number of cells invading Matrigel membrane/mean number of cells migrating through control membrane) ×100 (*P* < .01). (Upper) SB225002 significantly decreased the invasion of CXCL7-stable transfected MCF10AT cells compared with notreated cells. (Lower) Membranes were stained by Diff-Quik kit and were photographed (original magnification x 20).

To further verify CXCL7 function in cell invasion, a selective nonpeptide CXCR2 antagonist SB225002 [26] was used to check whether it can inhibit the invasive ability of MCF10AT cells transfected with CXCL7. MCF10AT cells were stably transfected with CXCL7 plasmid. After 48 hours of incubation, cells were added into the Matrigel invasion chamber; meanwhile, SB225002 (1.1 *μ*M) was also added into the chamber. Several concentrations of SB225002 were tested (0.5, 0.7, 0.9, 1.1, 1.3, and 1.5 *μ*M) and 1.1 *μ*M had the best inhibitory effect similar to that reported by Levashove et al. [23]. After 48 hours, cells were fixed and stained with Diff-Quick. The decrease in the percent invasion of Matrigel by the cells is shown in Figure 1. Treatment with SB225002 resulted in 18.06% ± 0.76% invasive cells compared with 55.7% ± 1.4% in nontreated CXCL7 stable transfected MCF10AT cells. Thus, the CXCR2 antagonist blocked the invasive ability of CXCL7 stably transfected MCF10AT cells.

### 3.2. Expression and Secretion of Lymphangiogenesis Factor VEGF-C and VEGF-D by Stable CXCL7 Transfected MCF10AT Cells Are Blocked by SB225002

**Figure 2:**
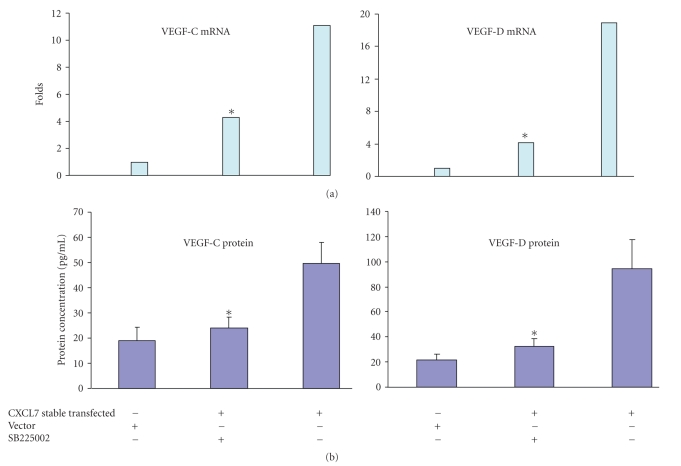
SB225005 blocked VEGF-C and VEG-D mRNA expression by CXCL7-transfected MCF10AT cells. CXCL7-stably transfected MCF10AT cells were cultured with and without SB225002 (1.1 *μ*M). After 48-hour treatment, the media and the cells were collected. (a) Quantitative analysis of VEGF-C and VEGF-D mRNA expression. Total RNA was extracted, and real-time qRT-PCR was performed. *β*-actin was applied as internal control. Triplicate determinations were performed. The differences between the cell lines were significant (*P* < .01)**. **(b) Quantitative analysis of VEGF-C and VEGF-D protein secretion by the CXCL7-stable transfected MCF10AT cells. Protein concentration in CM was measured by ELISA using human VEGF-C and VEGF-D ELISA Development System. Measurements were done at least in duplicate for 2 dilutions. The optical density of each well was determined using plate reader by subtracting the reading at 570 nm from the reading at 450 nm. VEGF-C/D content in CM of SB225002-treated group was significantly lower than untreated group (*P* < .05).

To investigate the effect of SB225002 on expression and secretion of VEGF-C and VEGF-D by stable CXCL7-transfected MCF10AT cells, we cultured the cells with and without SB225002 (1.1 *μ*M). After 48-hour treatment, the media and the cells were collected. Expression of mRNA for lymphangiogenic factors VEGF-C and VEGF-D was determined by quantitative real-time polymerase chain reaction (qRT-PCR) assay. The expression levels of mRNAs for VEGF-C, VEGF-D, are shown in Figure 2(a). *β*-actin was used as an internal control. Compared with vector-transfected MCF10AT cells, CXCL7-transfected MCF10AT cells showed 11-fold higher VEGF-C and 18-fold higher VEGF-D expression. Administration of SB225002 antagonist resulted in a significant (*P* < .01) inhibition of VEGF-C (3.5-fold) and VEGF-D (3-fold) mRNA expression. Next, we examined the secretion of the VEGF-C and VEGF-D by ELISA assay. The secretion levels of VEGF-C, VEGF-D are shown in Figure 2(b). Both VEGF-C and VEGF-D secretion from the CXCL7-transfected cells were significantly (*P* < .01) increased compared with the vector-transfected control group (VEGF-C, 3-fold; VEFG-D, 2.5-fold). Our data also showed a significant (*P* < .05) inhibition in VEGF-C and VEGF-D secretion by SB225002-treated cells compared with control treated cells. Thus the CXCR2 antagonist decreases VEGF-C and VEGF-D mRNA and protein expression.

### 3.3. CXCL7 siRNA Inhibited Heparanase Activity and Invasion of CXCL7 Stably Transfected Cells

**Figure 3:**
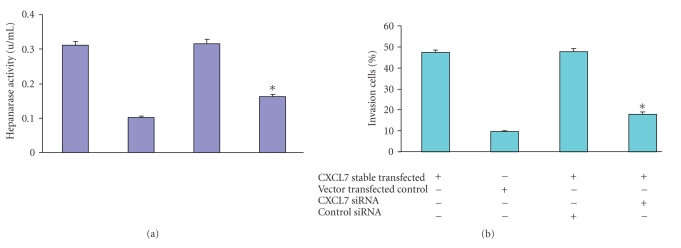
CXCL7 siRNA decreased heparanase activity and invasion of CXCL7-stably transfected cells. The CXCL7 MISSION siRNA was used to effectively knockdown heparanase activity and the invasion of CXCL7-stably transfected cells while the nontargeting siRNA was used as a control. CXCL7-stable transfected MCF10AT cells (2 × 10^5^) were transfected with 100 pmol of each siRNA by using the Lipofectamine 2000 transfection reagent (Invitrogen) after 48 hours in culture. (a) Quantifying heparanase activity. The CM was collected 24 hours later and analyzed by heparanase-degrading enzyme assay kit. Indicated amounts of cell lysate were incubated with biotinylated heparan sulfate at 37°C for 45 minutes, and enzyme activity was determined using an ELISA-type assay. Color was developed using the substrate supplied in the kit, and plates were read at 450 nm using a microplate reader. Decreased heparanase activity in CXCL7 siRNA. (b) Invasion of Matrigel. Using a BD BioCoat Matrigel invasion assay (6-well plates), 1.25 × 10^5^ cells/ml were inoculated onto the membrane in the upper chamber in serum-free medium. The lower chamber contained complete medium, and control membrane did not have Matrigel to measure migration of cells. Triplicate assays per group were completed. Results reported per cell line as % invasion = (mean number of cells invading Matrigel membrane/mean number of cells migrating through control membrane) ×100. CXCL7 siRNA significantly inhibited the invasion of CXCL7 stable transfected MCF10AT cell, while the control siRNA did not. Results compared by one-way ANOVA (*P* < .01).

Next, we examined whether CXCL7 siRNA inhibited the elevated heparanase activity of CXCL7-stably transfected cells. Cells were transfected with CXCL7 siRNA or control siRNA, and after 48 hours, the CM was collected. Compared with control siRNA treatment, CXCL7 siRNA-transfected cells showed significant (*P* < .01) inhibition of heparanase enzymatic activity (Figure 3(a)). The same results were obtained after using a Microcon filter to remove molecules less than 30 kD (which includes CXCL7) from CM, verifying by ELISA that CXCL7 is not present after filtering. And then, retesting for heparanase activity were performed.

To further confirm the increased invasion after transfection by CXCL7, we used the CXCL7 siRNA to block the CXCL7 signal. Matrigel invasion assays were performed with cells transfected with CXCL7 siRNA or control siRNA. The decrease in the percent invasion of Matrigel by the cells is shown in Figure 3(b). Treatment with siRNA resulted in 16% ± 1.2% invasive cells compared with 37% ± 2.5% by MCF10AT cells transfected with CXCL7. Thus, CXCL7 siRNA inhibits the invasion of MCF10AT cells transfected with CXCL7.

### 3.4. Expression and Secretion of Lymphangiogenesis Factor VEGF-C and VEGF-D by Stable CXCL7 Transfected MCF10AT Cells Are Silenced by sh-PPBP Transfection

**Figure 4:**
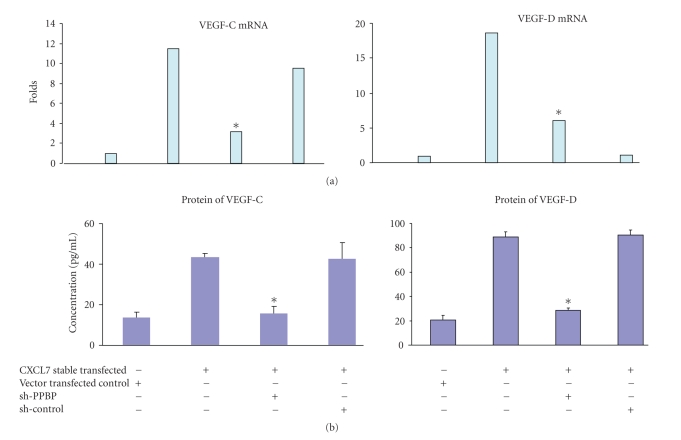
The sh-PPBP suppress VEGF-C and VEGF-D mRNA expression and secretion by CXCL7-transfected MCF10AT cells. We cultured the cells transfected with sh-PPBP or control sh-con. After 48 hours treatment, the media and the cells were collected. (a) Quantitative analysis of VEGF-C and VEGF-D mRNA expression. Total RNA was extracted, and real-time qRT-PCR was performed. *β*-actin was applied as internal control. Triplicate determinations were performed. The differences between the cell lines were significant (*P* < .01). (b) Quantitative analysis of VEGF-C and VEGF-D protein secretion by the CXCL7 stable transfected MCF10AT cells. Protein concentration in CM was measured by ELISA using human VEGF-C and VEGF-D ELISA Development System. Measurements were done at least in duplicate for 2 dilutions. The optical density of each well was determined using plate reader by subtracting the reading at 570 nm from the reading at 450 nm. VEGF-C and VEGF-D content in CM of sh-PPBP-transfected group was significantly lower than the control group (*P* < .05).

To further confirm the increased expression and secretion of lymphangiogenesis factors VEGF-C and VEGF-D after transfection by CXCL7, we used the sh-PPBP to silence the CXCL7 signal. To investigate the effect of sh-PPBP on expression and secretion of VEGF-C, VEGF-D of stable CXCL7-transfected MCF10AT cells, we cultured the cells transfected with sh-PPBP or control (sh-con). After 48 hours of transfection, the media and the cells were collected. Expression of the mRNA of lymphangiogenic factors VEGF-C and VEGF-D was determined by quantitative real-time polymerase chain reaction (qRT-PCR) assay. The expression levels of mRNAs for VEGF-C, VEGF-D, are shown in Figure 4(a). *β*-actin was used as an internal control. Compared with vector-transfected MCF10AT cells, CXCL7-transfected MCF10AT cells showed 11-fold higher VEGF-C and 18-fold higher VEGF-D expression. The sh-PPBP transfection resulted in a significant (*P* < .01) silencing of VEGF-C (66%) and VEGF-D (68%) mRNA expression. Next, we examined the secretion of the VEGF-C and VEGF-D by ELISA assay. The secretion levels of VEGF-C, VEGF-D are shown in Figure 4(b). Both VEGF-C and VEGF-D secretion from the CXCL7-transfected cells were significantly (*P* < .01) increased compared with the vector-transfected control group (VEGF-C, 3.3-fold; VEFG-D, 4.4-fold). Our data also showed a significant (*P* < .05) silencing in VEGF-C (65%) and VEGF-D (68%) secretion by sh-PPBP transfected cells compared with sh-con transfected cells.

### 3.5. ERK1/2 Mitogen-Activated Protein Kinase Pathway Is not Involved in CXCL7-CXCR2-Mediated Stimulation of Lymphangiogenic Factors VEGF-C, VEGF-D in Human Breast Cancer Cells

**Figure 5:**
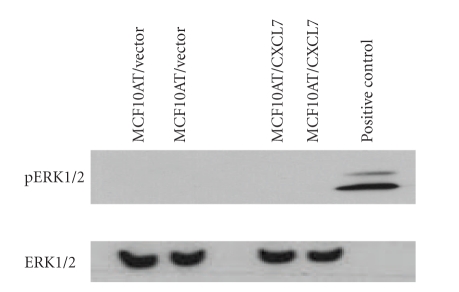
ERK1/2 mitogen-activated protein kinase is not active in CXCL7 stable transfected MCF10AT cells. Briefly, the cells were harvested in PBS, counted and lysed in the RIPA buffer with protease inhibitor cocktail. Protein concentration was determined for all samples using the Bio-Rad protein assay. The equal-volume samples (50 *μ*g) were separated by SDS-PAGE on a 10% polyacrylamide gel and transferred onto nitrocellulose membrane. Immunodetection was performed using pERK1/2 and ERK1/2, then developed by ECL. Stable CXCL7 transfected MCF10AT cells did not induce ERK1/2 phosphorylation compared to the vector transfected control cells. Furthermore, the expression of ERK1/2 was the same in stable CXCL7 transfected MCF10AT cells compared to vector controls.

The MAP kinase pathway is important for growth, differentiation, and migration, and is considered a dominant signaling pathway for CXCR2 [27, 28]. To investigate the mechanism by which the CXCL7-CXCR2 axis is involved in the stimulation of lymphangiogenic factors VEGF-C, VEGF-D toward invasion by human breast cancer cells, we investigated the involvement of ERK1/2 MAP kinases by western blotting. Stable CXCL7-transfected MCF10AT cells did not induce ERK1/2 phosphorylation in comparison with the vector transfected control cells, and there is no difference in ERK1/ 2 MAP kinase expression in stable CXCL7 transfected MCF10AT cells compared to vector controls (Figure 5).

## 4. Discussion

Although many molecules have been implicated in cancer metastasis, the detailed mechanism of tumor metastasis is still not completely understood. Recently, the interest in chemokines in cancer research has been increasing as new chemokines are being identified and investigated. Working along with many other molecules, the chemokines and their receptors expressed by both the cancer and its stroma influence growth, dormancy, angiogenesis, and invasion [3, 6, 29]. The CXCR4/CXCL12 (SDF-1) axis was the most common interaction that has been shown to be involved in many different human malignancies, including breast cancer, ovarian cancer, and prostate cancer [30, 31]. However, CXCR4 interactions alone did not completely explain the pattern of metastasis of cancer.

There are 4 families of chemokines (C, CC, CXC, and CX3C) based on the arrangement of cysteine residues near the N terminus [32, 33]. The CXC group can be further subdivided on the basis of presence of the tripeptide motif glutamate-leucine-arginine (ELR) adjacent to the CXC motif. All ELR^+^-CXC chemokines act through CXC chemokine receptor type 1 or type 2 (CXCR1 or CXCR2), which are transmembrane G-protein-coupled receptors [32]. ELR^+^-CXC chemokines can stimulate angiogenesis. Those without the ELR motif inhibit angiogenesis [34, 35]. Secretion of stromal cell-derived factor-1 (SDF-1)/CXCL12 and expression of CXCR4 have been identified to be associated with breast cancer metastasis [36]. Although CXCR4 was more highly expressed in the breast cancer cells tested byMüller et al.[37] than the other CXCRs, the expression of CXCR2 was also increased compared with the remaining receptors. CXCL7, which is a member of the ELR-CXC chemokines, binds with CXCR2 receptors, stimulating angiogenesis and association with neutrophils and other immune components [38]. Inhibition of CXCR2 function on endothelial cells has been shown to inhibit tumor growth, for lung cancer and renal cell cancer models [39, 40].

The use of a small selective antagonist for CXCR2 (SB225002) represents an attractive targeted therapeutic approach [26]. Previous work in our laboratory showed that breast cancer cells coexpress CXCL7 and CXCR2, which may act as a potential autocrine mechanism in breast cancer. The malignant cell line MCF10CA1a.cl1 strongly expressed CXCL7 and has much higher invasive ability than MCF10AT. MCF10AT cells gained invasive ability after they were transfected with CXCL7. Thus, CXCL7-transfected MCF10AT cells were as invasive as malignant cells, suggesting that CXCL7 may have a role in the invasion process. Therefore, targeting its receptor, CXCR2, seemed an obvious choice. 

 By using CXCL7-stably transfected MCF10AT cells treated by SB225002, invasion was decreased significantly compared with CXCL7-transfected cells, which might involve CXCL7 autocrine activity with CXCR2. The present study did not examine whether SB225002 could block the invasive ability of isogenic malignant cells (MCF10CA1a.cl1) or other malignant cell lines, which would be an interesting focus for future study.

Lymphangiogenesis refers to the formation of new lymphatic vessels that may occur in normal developing tissues or in tumors. Overexpression of VEGF-C or VEGF-D can lead to lymphangiogenesis, intralymphatic tumor growth and formation of lymph node metastases [41, 42]. VEGF-C and VEGF-D are ligands for VEGFR-3 (also termed fms-like tyrosine kinase 4, Flt-4), a tyrosine kinase receptor that is expressed predominantly in lymphatic endothelial cells [43]. The breast cancer cells secrete VEGF-C, VEGF-D which directly interact with the receptor. This paracrine relationship may lead to further changes in the breast cancer cells leading to invasion. Chemokine and VEGF-C interactions have been reported in a model of cross-talk for lymphatic endothelial cells and melanoma cells [4]. In our study, both the selective CXCR2 antagonist and sh-PPBP suppresses the elevated expression and secretion of VEGFC/D by the CXCL7-transfected MCF10AT cells. These results support the notion that the CXCL7/CXCR2 axis plays an important role in cancer cell lymphangiogenesis.

The chemokine connective tissue-activating peptide (CTAP-III), which is an N-truncated derivative of CXCL7, has been reported to have heparanase activity [15, 44]. The heparanase activity of chemokines may be important for modification of matrix [15]. Heparanase activity is also responsible for the egress of metastatic tumor cells and other blood-borne cells from the vasculature. Inhibition of heparanase activity results in decreased metastasis. Recently, expression of VEGF-C was shown to be induced by heparanase in prostate cancer cells, epidermoid cancer cells, breast cancer cells, and melanoma [6]. In this study, heparanase activity in the CM of CXCL7-transfected MCF10AT cells was tested, and it was determined that the CXCL7-transfected cells demonstrated increased secreted heparanase activity. This elevated activity was inhibited by CXCL7 siRNA. In addition, silencing CXCL7 inhibited the invasive ability of CXCL7-transfected MCF10 AT cells which further elucidates its role in invasion. The expression of VEGF-C and VEGF-D mRNA and protein was also decreased in these transfectants by shRNA, thus linking the expression of the chemokine CXCL7 to VEGF-C and VEGF-D, heparanase expression, and invasive ability. The MCF10 model of progressive breast disease provides isogenic cell lines with increasing malignant potential to study the steps of metastasis. Additional breast cancer cell lines and breast cancer tissue can be tested specifically for CXCL7 expression and effects on lymphangiogenesis, heparanase expression and invasion in the future. Lymphangiogenesis in human breast cancer samples can be correlated with clinical parameters and CXCL7/CXCR2 staining.

To explain the mechanism by which the CXCL7-CXCR2 axis is involved in the stimulation of lymphangiogenic factors VEGF-C, VEGF-D, increased invasion and heparanase expression in human breast cancer cells. We investigated the MAP kinase signaling pathway [45, 46]. We demonstrated that CXCL7-stable transfected MCF10AT cells do not activate ERK1/2 MAP kinase signaling. We did not observe a difference of ERK1/2 kinase in CXCL7-stable transfected MCF10AT cells compared with control cells. This result suggests the involvement of different pathways other than MAP kinase signaling for the CXCL7-CXCR2 axis activation. In general, the activation of G-protein coupled receptors (GPCRs) like CXCR2 leads to the dissociation of the *α* subunit from the *β*,*γ*-dimer when GDP is replaced by GTP [47]. Both subunits can activate many signaling pathways including phospholipase C and adenyl cyclase. When specifically considering CXCR2, information from CXCL8 might shed some light on other potential pathways for CXCL7 since these two chemokines have 48% identity in their amino acid sequences and both bind CXCR2 [48]. CXCL8 binding to CXCR2 activates the Rac/PI3K, Rho and Ras pathways [49]. Therefore, the CXCL7/CXCR2 axis may activate pathways other than the MAP kinase pathway, including Rac/PI3K, Rho and Ras pathways.

## 5. Conclusions

 In the present paper we showed that both the selective CXCR2 antagonist SB225002 and sh-PPBP suppress VEGF-C, VEGF-D expression and secretion in CXCL7-transfected MCF10AT cells. Furthermore, we also observed that both SB225002 and CXCL7 siRNA reduced the invasion of CXCL7-stably transfected MCF10AT cells, confirming the role of the CXCL7/ CXCR2 axis in cell invasion, possibly through the receptor's signaling mechanism. However, this does not involve the MAP kinase signaling pathway as has been described for other ELR^+^ CXC chemokines like IL-8. It has also been shown that CXCL7 siRNA knocked down heparanase activity in transfected MCF10AT cells, suggesting an important role for CXCL7 in heparanase expression. Taken together, these data would support that the CXCL7/CXCR2 axis may be important in breast cancer metastasis. Therapeutics aimed at antagonizing CXC chemokine action, including CXCL7, may be beneficial in preventing invasion and thus the spread of breast cancer.

## Figures and Tables

**Table 1:** Summary of primers for Real-Time PCR.

Gene: VEGF-C
GenBank Accession No.: NM_005429
Product size: 128 bp
Primers:
*Forward: *5′-GCCACGGCTTATGCAAGCAAAGAT-3′
*Reverse: *5′-AGTTGAGGTTGGCCTGTTCTCTGT -3′
Gene: VEGF-D
GenBank Accession No.: NM_004469
Product size: 132 bp
Primers:
*Forward: *5′-CGATGTGGTGGCTGTTGCAATGAA -3′
*Reverse: *5′-GCTGTTGGCAAGCACTTACAACCT -3′
Gene: beta-actin
GenBank Accession No.: X00351
Product size: 125 bp
Primers:
*Forward: *5′-GGACTTCGAGCAAGAGATGG-3′
*Reverse: *5′- AGCACTGTGTTGGCGTACAG -3′
